# Impacts of the Pandemic on Older Adults’ Social Isolation

**DOI:** 10.1093/geroni/igab046.3405

**Published:** 2021-12-17

**Authors:** Stephanie MacLeod

**Affiliations:** UnitedHealth Group, United States

## Abstract

The risk of COVID-19 exposure and likelihood of severe illness have been critical concerns among older adults during the pandemic. Meanwhile, social distancing has worsened social isolation, with severe impacts on connectedness among seniors. Effects of the pandemic may lead to an extended crisis, with impacts on health outcomes. Our primary purpose was to summarize emerging research describing impacts of the pandemic on social isolation and related health outcomes among older adults. A streamlined search was conducted to fit the scope of this review, with key terms determined to identify relevant publications. Common research databases and mainstream resources were utilized. We focused on research published or released since the start of 2020, primarily rapidly reviewed studies, to align with the timing of the pandemic. Early research suggests that the pandemic has worsened social isolation among older adults. Social isolation is now more urgent, as many seniors lost their usual connections due to social distancing. While these measures help to prevent virus exposure, this approach must be balanced with maintaining social connectedness. Thus, a “COVID-19 paradox” has emerged: safety protocols protect older adults but concurrently place them at risk of social isolation. Adapted approaches are urgently needed to safely address the consequences of a potential long-term social recession.

